# Concentrations of faecal glucocorticoid metabolites in Asian elephant's dung are stable for up to 8 h in a tropical environment

**DOI:** 10.1093/conphys/cow070

**Published:** 2016-12-29

**Authors:** Ee Phin Wong, Lisa Yon, Rebecca Purcell, Susan L. Walker, Nasharuddin Othman, Salman Saaban, Ahimsa Campos-Arceiz

**Affiliations:** 1 School of Environmental and Geographical Sciences, Faculty of Science, The University of Nottingham Malaysia Campus, Jalan Broga, Semenyih 43500, Kajang, Selangor, Malaysia; 2 School of Veterinary Medicine and Science, Faculty of Medical & Health Sciences, The University of Nottingham, Sutton Bonington, LeicestershireLE12 5RD, UK; 3 Chester Zoo, Upton-by Chester, ChesterCH2 1LH, UK; 4 Department of Wildlife and National Parks Peninsular Malaysia, Km. 10, Jalan Cheras, 56100 Kuala Lumpur, Malaysia; 5 Mindset, Interdisciplinary Centre for Tropical Environmental Studies, The University of Nottingham Malaysia Campus, Jalan Broga, Semenyih 43500, Kajang, Selangor, Malaysia

**Keywords:** Adrenal activity, Asian elephant, dung decay, *Elephas maximus*, faecal glucocorticoid metabolites, non-invasive monitoring

## Abstract

The use of faecal glucocorticoid metabolites (fGCMs) has facilitated the development of non-invasive methods to study physiological conditions of endangered wildlife populations. One limitation is that fGCM concentrations are known to change over time and to vary according to different environmental conditions. The aim of this study was to perform a controlled dung decay experiment to understand the impact of time (since defecation) and two common environmental variables (exposure to water and direct sunlight) on fGCM concentrations of Asian elephants (*Elephas maximus*). Eighty dung piles from 10 Malaysian elephants were randomly exposed to a 2 × 2 combination of treatments (wet–shade, dry–shade, wet–sun and dry–sun) and repeatedly subsampled from the time of defecation through to 2 days post-defecation (*n* = 685 faecal subsamples). Overall, the mean concentration of fGCMs was stable in samples of up to 8 h old from defecation time, regardless of environmental treatment (water or direct sunlight); thereafter, the overall mean fGCM concentrations increased, peaking 1 day after defecation (31.8% higher than at defecation time), and subsequently decreased (reaching values 9.2% below defecation time on the second day). Overall, the treatment of sun exposure resulted in higher fGCM concentration compared with shade, whereas water exposure (compared with no water exposure) had no impact on fGCM concentrations. Hence, in field studies we recommend collecting dung samples <8 h old and recording shade conditions (e.g. sun vs. shade) as a covariate for the subsequent interpretation of fGCM measurements. This study has helped to identify the optimal window for sampling in which we can have a higher confidence in interpreting the results as being a genuine reflection of glucocorticoid status in the elephant.

## Abstract (precis)

The concentration of faecal glucocorticoid metabolites in dung of Asian elephants in a semi-natural tropical rainforest of Malaysia was stable for up to 8 h and affected by exposure to sun but not to water. This information is key for the effective design of field studies of faecal glucocorticoid metabolites.

## Introduction

Faecal endocrinology has important applications for wildlife conservation because it facilitates the non-invasive monitoring of adrenal activity in wild animal populations (Wasser *et al*., 2000; Möstl and Palme, 2002; Sheriff *et al*., 2011; Watson *et al*., 2013). The concentration of faecal glucocorticoid metabolites (fGCMs) is a reliable indicator of biologically active (‘free’) glucocorticoid metabolites circulating in an animal's body over a period of time and, importantly, wildlife faeces are easier to collect than other biological samples, such as blood, saliva or urine (Möstl and Palme, 2002; Touma and Palme, 2005). Studies of fGCMs have been conducted to investigate a number of conservation-related questions for a range of wildlife species, including African elephants (*Loxodonta* sp.; Gobush *et al*., 2008; Viljoen *et al*., 2008; Pinter-Wollman *et al*., 2009; Ganswindt *et al*., 2010) and Asian elephants (*Elephas maximus*; Laws *et al*., 2007; Fanson *et al*., 2013; Watson *et al*., 2013).

The hormone metabolite concentration in a faecal sample can vary over time (e.g. in the time elapsed between defecation and sample collection by a researcher), and this variation is mediated by environmental factors such as ambient temperature and moisture (Millspaugh and Washburn, 2004, 2003) as well as by the effects of bacterial activity (e.g. Wasser *et al*., 2000; Möstl and Palme, 2002). For example, fGCM concentration rapidly became unstable over time in faeces of captive and wild orangutans (*Pongo pygmaeus*; Muehlenbein *et al*., 2012) and showed fluctuations in faeces of African savanna elephants (*Loxodonta africana*; Stead, 2000). Moreover, exposure to different temperatures and humidity treatments had complex effects on the fGCM concentration in faeces of white-tailed deer (*Odocoileus virginianus*; Washburn and Millspaugh, 2002), wild bears (*Ursus* spp.; Stetz *et al*., 2013) and jaguars (*Panthera onca*; Mesa-Cruz *et al*., 2014). These results highlight the importance of assessing fGCM stability in field conditions in studies using fGCM to monitor wildlife populations.

Asian elephants are the largest terrestrial animals in Southeast Asia and a species of great ecological significance (Campos-Arceiz and Blake, 2011; Campos-Arceiz *et al*., 2012) that have become endangered as a result of the rapid decline of their populations in recent decades (Choudhury *et al*., 2008). The conservation of Southeast Asian elephants is hampered by a poor understanding of their ecology and behaviour and the difficulty of studying them in tropical rainforests (Blake and Hedges, 2004). With the general aim of defining appropriate sampling protocols to study wild elephant populations in Southeast Asia, here we present a study to determine fGCM stability in semi-natural conditions in Peninsular Malaysia. Our objectives were as follows (i) to determine how long the fGCM concentration in Asian elephant faeces remains stable after defecation; and (ii) to determine whether fGCM stability is affected by exposure to sunlight and/or water. To address these questions, we conducted a dung decay experiment with elephants in semi-natural habitats in Peninsular Malaysia.

## Materials and methods

### Animals

Faecal samples were collected from 10 elephants: eight females (seven adults and one sub-adult) and two males (both sub-adults), from Kuala Gandah National Elephant Conservation Centre (NECC) in Pahang, Peninsular Malaysia. Elephants in our sample were all older than 10 years; we classified as sub-adults those with an estimated age of <15 years. All the elephants were housed in the same paddock area at night, in similar environmental conditions. The elephants were fed a consistent diet of grasses supplemented with papaya and sugarcane; during the day, they were also allowed to graze on different patches of grasslands. At noon, elephants were given a daily bath in a river.

### Collection of faecal samples

The dung piles (*n* = 80, three to nine boli each) were collected from the 10 study individuals for five consecutive days (13–17 January 2013) between 04.00 and 09.00 h. Following defecation by any of the elephants, the dung piles were collected and sampled immediately (hereafter known as ‘time 0’ samples) before being randomly assigned to one of the 2 × 2 treatment combinations [wet–shade (*n* = 21 faecal piles), wet–sun (*n* = 20), dry–shade (*n* = 17) and dry–sun (*n* = 22)]. For ‘wet’ samples, 1 litre of purified water was poured over the dung pile just after the time 0 sample was taken; for ‘dry’ samples, no water was added; for ‘shade’ samples, the dung piles were placed in an area under a tree canopy (70–100% canopy cover); and for ‘sun’ samples, the dung piles were placed in an open field (0% canopy cover). At night (19.00–04.00 h) and during two brief episodes of rain, we covered all the samples with plastic sheets (placed over each dung pile) to avoid rainfall affecting our experimental treatments. During the experiment, the nearest weather station (Felda PPP Tun Razak, 03°50′N, 102°34′E) recorded daily averages of 6.4 ± 2.5 h of sunlight, 25.5°C temperature, 4 ± 8 mm of rainfall and 80 ± 2% relative humidity.

To collect faecal subsamples (*n* = 685 from the 80 dung piles) for hormone analysis, we used scissors to cut three small openings in different parts of the dung pile (usually on different boli) and removed faecal matter from the centre of the bolus using forceps; we then placed the sample in a ziplock bag, mixed thoroughly, and kept it frozen at −20°C until laboratory analysis. Each dung pile was coded with a unique identity (ID) number. Hormone sample bags were labelled with dung pile ID, date and time of sampling. Faecal subsamples were taken at a range of times, with sampling occurring more intensively within the first half of the day of defecation to capture initial changes in fGCM and less frequently thereafter, as follows: time 0 (fresh defecation), 0–2, 2–4, 4–6, 6–8, 8–11 and 11–16 h and 1, 1.5 and 2 days after defecation. Dung piles collected at the start of the day (e.g. at 04.00 h) were sub-sampled more frequently than dung piles collected later in the day (e.g. at 09.00 h), hence the number of subsamples differed between dung piles.

### Hormone analysis in the laboratory

The faecal subsamples were extracted using a wet-weight extraction technique adapted from Walker *et al*. (2002) and described elsewhere (Watson *et al*., 2013; Edwards *et al*., 2014), whereby 0.5 g of faecal matter was extracted with 5 ml of 90% methanol, shaken overnight, dried and reconstituted in 1 ml of 100% methanol, and stored at −20°C until being analysed with a corticosterone enzyme immunoassay (CJM006; supplied by Coralie Munro, University of California Davis, CA, USA). The assay has been biologically and biochemically validated for use in Asian elephants (Watson *et al*., 2013). The corticosterone antiserum CJM006 cross-reactivities are published elsewhere (Watson *et al*., 2013), and only data with an intra-assay coefficient of variation of <10% and inter-assay coefficient of variation of <15% were accepted and used for statistical analysis.

### Data analysis

The fGCM concentrations were log_10_ transformed and analysed using cross-classified linear mixed models to determine relevant random effects and assess the effect of the fixed factor treatments. One dung pile was removed from the data set and subsequent analyses because it had been sampled fewer than four times during the study, and three other dung piles were removed because they were considered to contain some extreme outliers (fGCM concentration >80 ng/g). The removal of these dung piles did not affect the outcome of the final model selection. The final data set, therefore, included data from 76 faecal piles with a total of 660 subsamples. The fixed factors of interest were as follows: (i) time since defecation, as a categorical variable [time 0 (fresh defecation), 0–2, 2–4, 4–6, 6–8, 8–11 and 11–16 h and 1, 1.5 and 2 days after defecation]; (ii) water (wet vs. dry); (iii) shade (shade vs. sun); and (iv) the interaction between water and shade. The random factors considered were as follows: (i) variation within elephant individuals (*n* = 10); (ii) variation within dung piles (*n* = 76); and (iii) variation within days of collection (*n* = 5). The variation within dung piles was considered to be nested within the variation of individual elephants.

We used time as a categorical variable instead of a continuous variable (in quadratic form) to make it easier to detect the point in time at which fGCM became different in comparison to time 0. To test the effect of time since defecation on fGCM concentration, we set the contrast to compare all time points against time 0 values.

All linear mixed models were fitted using the function lmer in the package lme4 (Bates *et al*., 2015) in the R statistical environment (version 3.1.1; R Core Team, 2014). The optimal model was determined by dropping of the variables in the model one by one and comparing its significance with the model using likelihood ratio (LR) tests (Galecki and Burzykowski, 2013; Zuur *et al*., 2009). First, to choose the random covariates to retain in the final model, a restricted estimated maximum likelihood and combination of LR tests, Akaike information criterion and Bayesian information criterion was used (Zuur *et al*., 2009). The effects of the fixed factors were assessed on the model using maximum likelihood by dropping the fixed factors one at a time and comparing the new model with the previous one. Again, we kept the fixed factors that were significant for the model based on LR tests and information from the Akaike information criterion and Bayesian information criterion.

We used a supporting R package, lmerTest (Kuznetsova *et al*., 2015) with Satterthwaite approximation to estimate degrees of freedom to calculate *P*-values and confidence intervals for the model parameters. Density plots from simulated models were obtained (repeated 1000 values), which plotted the effect of fixed factors and random effects in the model for comparison (Orelien and Edwards, 2008; Galecki and Burzykowski, 2013). Finally, we checked the model's assumption of homogeneity and normality of residuals using Pearson standardized residuals.

## Results

**Figure 1:**
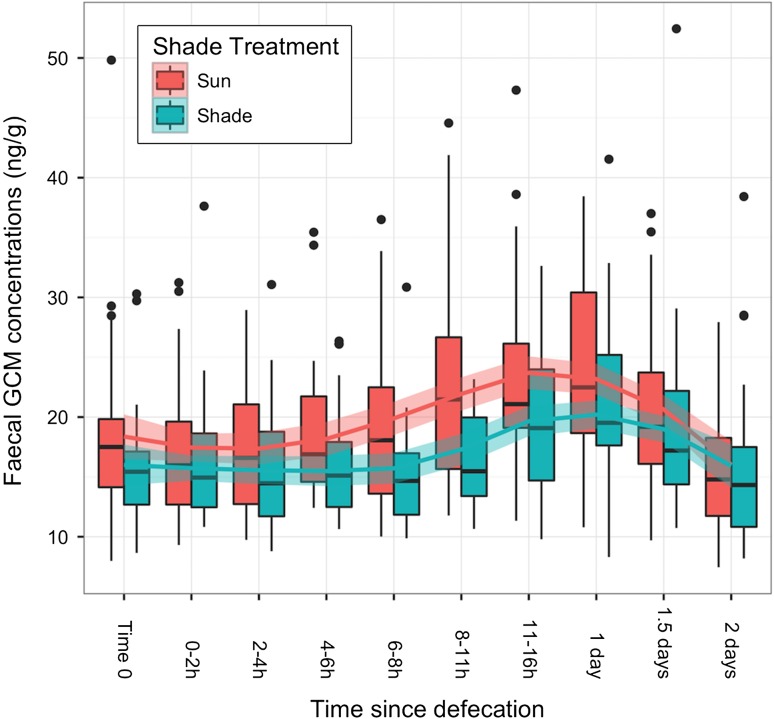
Changes in faecal glucocorticoid metabolite (fGCM) concentrations in Asian elephant dung (*n* = 76 dung piles, *n* = 660 faecal subsamples) from the time of defecation (time 0) to 2 days post-defecation. Samples under direct sun exposure (red) showed higher fGCM concentrations compared with samples under shade (green).

The overall mean ± SD concentration of fGCM at time 0 (fresh defecation) was 17.35 ± 6.23 ng/g, and it remained stable for up to 8 h after defecation, with fluctuations within ±6.0% from the time 0 mean (Fig. 1). After 8 h, the mean fGCM concentration increased considerably, being 13.20% higher at 8–11 h after defecation, 26.17% higher at 11–16 h, and reaching its peak 1 day after defecation at values 31.82% higher than time 0. Thereafter, the fGCM concentration decreased, showing values slightly higher (14.52%) than time 0 after 1.5 days and dropping below time 0 values (−9.16%; 15.76 ± 5.93 ng/g) 2 days after defecation (Fig. 1).

**Figure 2:**
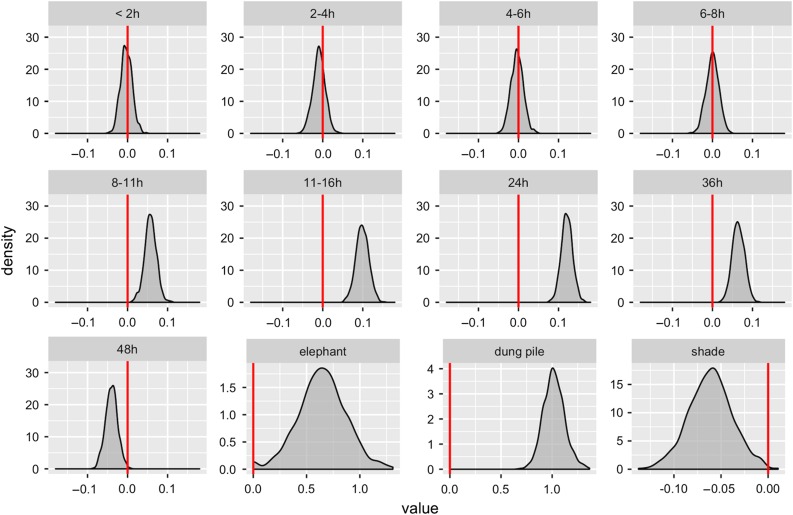
Density plots from simulated model data (*n* = 1000) were generated to estimate *P-*values for fixed and random effects. The value of fixed effect coefficient ‘β’ for each time sector compared with categorical time 0 (i.e. fresh defecation) and shade results is the difference of shade in comparison to ‘sun exposure’. If the β-value is centred around zero (red vertical line) then there little difference between the two categories; if the value is far from zero, then the difference between the two categories is large. In terms of changes of fGCM over time, this figure shows that the fGCM concentration remains stable for up to 8 h after defecation, but thereafter there are large differences from the time 0 baseline. The random effects ‘intercepts’ (dung piles and individual elephants) were compared with ‘intercept = 0’ and are significant for the model.

The analysis of random effects showed that individual dung piles (χ²_1_ = 255.4, adjusted *P* < 0.001) and individual elephants (χ²_1_ = 9.27, adjusted *P* < 0.001) were influential effects. The covariate ‘day of collection’ was not influential on the data and was dropped from the model (χ²_1_ = 0.00, adjusted *P* = 0.50; Fig. 2). The analysis for the fixed factors, time (χ²_1_ = 183.82, *P* < 0.001) and shade (shade vs. sun), had significant effects (χ²_1_ = 7.12, *P* = 0.008; Figs 1 and 2 and Table 1), with sun exposure resulting in higher fGCM concentration compared with shade exposure (Fig. 1). The treatment of water did not have a significant effect on fCGM concentrations (χ²_1_ = 0.18, *P* = 0.67). Additionally, the interactions between (i) water, shade, and time; (ii) water and time; (iii) shade and time; and (iv) water and shade treatments had no impact on fGCM concentration (*P* > 0.05). The final linear mixed models found that time categories <8 h were not significant (*P* ≥ 0.54), whereas categories >8 h were significantly different from time 0 (*P* < 0.001; Fig. 1). The results of the final model (Table 1) were confirmed by the model simulation, which demonstrated that fGCM concentration values remained stable for up to 8 h after defecation but not afterwards, and supported our retention of elephant and dung pile (nested within elephant) as random effects and shade as a significant fixed factor.

**Table 1:** Results from the final model, with *P*-values for the fixed factors and confidence intervals for intercepts and slope (β coefficient) generated from lme4 and lmerTest package

Type	Factor	Coefficent (SE)	d.f.	*t*-value	95% Confidence interval	*P*-value
Fixed	Intercept	1.244 (0.026)	17.70	47.71	1.193–1.297	<0.00001
	Shade	−0.039 (0.022)	68.0	−2.73	−0.106 to −0.017	0.008
	<2 h	−0.061 (0.014)	580.4	−0.21	−0.031 to 0.025	0.83
	2–4 h	−0.0031 (0.015)	577.9	−0.62	−0.040 to 0.021	0.54
	4–6 h	−0.009 (0.016)	577.7	−0.17	−0.033 to 0.028	0.86
	6–8 h	−0.002 (0.016)	579.6	0.01	−0.030 to 0.031	0.99
	8–11 h	0.000 (0.016)	577.4	3.75	0.028 to 0.088	0.0002
	11–16 h	0.056 (0.016)	579.7	6.15	0.067–0.130	0.00001
	1 day	0.099 (0.015)	575.4	8.38	0.093–0.150	0.00001
	1.5 days	0.122 (0.015)	576.2	4.37	0.036–0.095	0.00001
	2.0 days	0.066 (0.015)	575.7	−2.65	−0.068 to 0.010	0.008
Random	Elephant	0.056			0.009–0.093	<0.001
	Dung pile	0.089			0.071–0.109	
	Random effect residual	0.086			0.084–0.094	

The parameters for random factors were obtained through simulation (×1000) by comparing the final model with both random factors and null model without random factors.

## Discussion

We found that fGCM concentrations in Asian elephants’ faeces were stable for up to 8 h in semi-natural conditions in the rainforests of Southeast Asia, hence allowing us to design a reliable protocol for dung sampling for the monitoring of adrenal activity in wild elephants. This is especially relevant when tracking wild elephants with GPS satellite collars in the thick forest. In the event that the observer could not determine the exact moment when the elephant defecates, the age of the dung pile can be estimated conservatively based on when the elephant was in a particular location.

The large sample size from this study provides confidence in our results regarding the changes in fGCMs that occurred in elephant faeces up to 2 days old. It is important to note that the fGCM concentration became elevated after 8 h, and more rapidly when exposed to direct sunlight. The previous performance (biological validation) of this assay on captive Asian elephants suggests that fGCM concentrations >25 ng/g reflect elevated glucocorticoid concentrations associated with a significant challenge (Watson *et al*., 2013). If the effect of time elapsed since defecation is ignored in a field study, researchers might erroneously conclude that a particular animal has elevated adrenal activity when it is in fact an artefact of the sample's age or of exposure to environmental conditions. Future field studies should therefore consider dung decay in their study design to determine the suitable window for sample collection, as well as interpreting and comparing fGCM results separately for faecal samples collected in shaded locations and those collected in open areas directly exposed to the sun.

The biological mechanisms that influence the changes in fGCMs over time could be caused by bacterial activity or transformation and breakdown of the glucocorticoid's molecular structure, resulting in differences in affinity with the antibody in the assay (Palme, 2005). Steroid hormones in the environment can be removed from the environment through sorption (absorption and adsorption), photolytic degradation (chemical decomposition in sunlight) or microbial activity (Snow *et al*., 2013). For example, in a loam soil sample mixed with 80% sterilized soil, the degradation of 17β-estradiol to estrone was much slower than in fully unsterilized soil, implying that soil microorganisms play an important role in the degradation of steroid hormones (Xuan *et al*., 2008; Snow *et al*., 2009).

It is not clear why water exposure resulted in an elevation in fGCM concentrations in some studies (Washburn and Millspaugh, 2002; Mesa-Cruz *et al*., 2014) but not in the present study. Some possible reasons are as follows: (i) water might cause changes in metabolite immunoactivity that can be picked up by some enzyme immunoassays or radioimmunoassays but not by others (Mesa-Cruz *et al*., 2014); (ii) there could be differences in faecal forms (e.g. elephant dung is voluminous and highly fibrous, bird faeces are in the form of powder, and deer faeces are small dense pellets) or other unmeasured variables that might be influencing the interaction between water and the metabolism of the glucocorticoids present in the faeces; (iii) the high humidity in the tropical environment might also affect samples not treated with water and thus confound the effect of the water treatment; and (iv) it is possible that the amount of water used in our experiment was insufficient to affect fGCM concentrations. Given that elephant dung is full of fibre, it contains natural moisture and it has a protective mucus on the outer layer, so the addition of water might not have a major influence on the fGCMs in the centre of the boli, from where the samples were taken. In the present study, we used purified water as a proxy for rainwater. We acknowledge that there are chemical differences between both and recommend that future studies should use forest rainwater to investigate the effect of rain on fGCM stability.

Although we conducted this experiment with captive elephants, whose diet and daily activities differ from those of wild individuals, we think that our results are relevant for studies on wild elephants. Similar studies would be impossible to carry out using dung from wild elephants in the tropical rainforests of Southeast Asia because in these environments: (i) elephants occur at low densities; (ii) direct observation is unfeasible; and (iii) getting near elephants to collect freshly defecated dung would involve a high physical risk for the researchers. The NECC has the biggest captive elephant population in Peninsular Malaysia, and we were able to have access to eight females (65 dung piles) and two male elephants (15 dung piles) in the present study. Although this sample size is adequate to study fluctuations in fGCM in individual dung piles over time, future studies could look into individual variability in fGCM in relationship to ‘stress treatments’ (see contextual interpretation of fGCM; Madliger and Love, 2014) and try to sample from a larger population with equal representation from different age groups and sex. Overall, our results have important implications for studies of free-ranging wildlife, highlighting that the time since defecation and the effect of environmental factors affect fGCM concentration; hence, they should be important considerations in field fGCM sampling protocol design.
